# Pulmonary arterial pressure in at-term in vitro fertilization neonates: A cross-sectional study

**DOI:** 10.4274/tjod.galenos.2020.74152

**Published:** 2020-07-29

**Authors:** Mohammad Reza Alipour, Zohreh Pezeshkpour, Seyedeh Mahdieh Namayandeh, Mohammadtaghi Sarebanhassanabadi

**Affiliations:** 1 Shahid Sadoughi University of Medical Sciences, Yazd Cardiovascular Research Center, Yazd, Iran

**Keywords:** In vitro fertilization, persistent pulmonary hypertension, neonate

## Abstract

**Objective:**

Hormones consumption in women who conceive through in vitro fertilization (IVF) as well as embryonic manipulations have raised concerns regarding the neonates’ health, including the possibility of pulmonary hypertension. This study, therefore, aimed to assess the pulmonary arterial pressure in at-term IVF neonates.

**Materials and Methods:**

This prospective cross-sectional study was conducted between March 2013 and October 2017 and compares 160 IVF neonates (group 1) with 160 naturally conceived neonates (group 2). The neonates in both groups were cesarean newborns, matched in terms of gestational and neonatal age. The neonates were three-seven days old, had a full-term gestational age of 37-39 weeks and 6 days, and a normal birth weight of 2500-4000 gr. The systolic pulmonary artery pressure (SPAP) was estimated using real-time echocardiography on the basis of peak flow velocity of tricuspid regurgitation jet.

**Results:**

A significant difference was observed in the mean SPAPs between the two groups (p<0.001). Although, the effect of gestational age on reducing SPAP was greater and statistically significant in group 1, the gradual decrease in the PAP after birth appeared to be slower in this group. Moreover, in both groups, the effect of gestational age on reducing SPAP was more convincing than that of the neonatal age. Further, in both groups, a significant reverse correlation was observed between the SPAP and the neonatal weight; however, it appeared to be markedly higher in group 1.

**Conclusion:**

Our study renders IVF as being culpable in the incidence of pulmonary hypertension among neonates. Hence, to detect the likelihood of pulmonary arterial hypertension in IVF neonates, it is recommended to monitor their PAP during the neonatal period, and thereby facilitate them with the required treatment.

**PRECIS:** The pulmonary arterial pressure in at-term in vitro fertilization neonates.

## Introduction

As the fetus leaves the uterus, the pulmonary arterial pressure (PAP) starts to drop, and as a result, the vascular resistance of the lungs begins to decrease. Due to the gradual dilatation of the neonates’ pulmonary arterioles lining, it usually reaches that of an adult within a week, or a maximum of six-eight weeks^(1)^.

Pulmonary hypertension results from a lack of proper decrease in the vascular resistance of the lungs after the neonatal period^(2)^, usually at term or later up to 34 weeks^(3)^. The prevalence rate of this lung condition is approximately 2 in 1,000 live births^(2)^ with a mortality rate of about 4%-33%^(3)^. Arterial pulmonary hypertension is a critical and severe progressive status with weak prognosis^(4)^, and the likelihood of the survival amounts to around 69% following the conventional treatments^(5)^.

The method of delivery, especially, cesarean section, may also indirectly raise the risk of pulmonary hypertension as it limits the synthesis of pulmonary endogenous vasodilators, lowers the level of protective antioxidants in newborns, and exposes them to a higher risk of respiratory distress syndrome and elevated level of endothelin-1^(6)^.

On the other end of the spectrum, *in vitro* fertilization (IVF) has been in use for three decades, so much so that assisted reproductive technology (ART) children account for about 1%-4% of births in the developed countries^(7)^. Although, the consumption of different hormones by women as well as the embryonic manipulations have raised many concerns regarding the neonates’ health, including the possibility of pulmonary hypertension, yet, the issue remains to be further addressed. Therefore, the aim of this study was to investigate the effect of the type of pregnancy-IVF in this study-on the blood pressure of the pulmonary artery.

## Materials and Methods

This study is a prospective cross-sectional study that was conducted between January 2011 and August 2019 and compares 160 neonates conceived through IVF (group 1) with 160 neonates conceived naturally (group 2).

In order to control the type of delivery as a confounding factor^(6)^, all subjects (in both groups) were selected from a cesarean section population. Moreover, to eliminate other confounding factors such as gestational and neonatal age, both groups were matched using the individual matching method. First, 160 IVF neonates born through cesarean section were admitted into the study (group 1), then, 160 others who were born through cesarean section but gestated naturally were matched with the first group for gestational and neonatal age, and all of them were included into the study.

Mothers with a history of premature rupture of the membranes, gestational infection, diabetes, or other underlying conditions were excluded from the probe^(2)^.

The neonates were about three-seven days old, all being at term with a gestational age of 37-39 weeks and 6 days, and had a normal birth weight of 2500-4000 gr.

To estimate the systolic pulmonary artery pressure (SPAP), a real-time phased-array sector scanner echocardiography with a Color Doppler echocardiograph, the Vivid 3 expert model (GE Healthcare, USA) version 2011, was used with an integrated Color Doppler system and transducer containing crystal sets for two-dimensional image (5.0 MHz with second harmonic technology) and continuous-wave Doppler recorder (2.5 MHz).

When tricuspid regurgitation was localized with color-flow Doppler, the peak flow velocity of tricuspid jet was measured using a continuous-wave Doppler. The pressure gradient between the right ventricle (RV) and the right atrium (RA) was calculated using modified Bernoulli’s equation (10 and 11) at least thrice for each neonate and the mean was then recorded. Note that this approach, that is, measuring the pressure gradient between RV and RA, is a non-invasive standard method for estimating SPAP^(8)^.

Based, on this echocardiography the neonates with a mean pulmonary arterial pressure (MPAP) >25 mmHg (SPAP > 36 mmHg) were considered as pulmonary hypertensive^(1)^.

As a sub-target, the presence of patent ductus arteriosus (PDA) was also examined during the echocardiography using color-flow Doppler and continuous-wave Doppler.

This study was approved by the Ethical Committee of Yazd, Iran Yazd Cardiovascular Research Center (approval number: 814). Informed consent was obtained from the participants.

### Statistical Analysis

Finally, data were analyzed through the SPSS software version 19 using the following tests: t-test for continuous quantitative variables, Fisher’s exact test for comparing variables between ART and the control group, and analysis of variance for comparing the two groups.

## Results

No significant difference was observed in the mean gestational age, neonatal age and weight, as well as the mean age of the mothers in both groups. Also, the gender proportion appeared to be similar in both groups (Table 1).

The number of boys in groups 1 and 2 were 85 (53.1%) and 75 (46.9%), respectively, therefore, no significant difference was observed between the two groups in terms of gender (p=0.15).

While the mean SPAP in group 1 was 28.06+-4 mmHg, it was 22.05+-5 mmHg in group 2, thus being 27.25% higher and statistically significant (p<0.0001).

The relationship between SPAP and gestational age in group 1 was also significant but waned with growing gestational age of SPAP. In addition, while the relationship projected to be significantly higher in group 2, the relationship was more convincing in group 1. The correlation coefficient (r) turned out to be -0.74 and -0.39 in groups 1 and 2, respectively. Moreover, linear regression (B) between SPAP and gestational age performed in both groups revealed that in group 1, with a daily increment in gestational age, SPAP diminished up to 0.98 mmHg. However, in group 2, a daily rise in gestational age lowered SPAP up to 0.45 mmHg (Graphic 1).

A comparison of SPAP with neonatal age in group 1 revealed an inverse and significant correlation between these two variables, that is, with a daily increase in neonatal age, SPAP dwindled up to 0.19 mmHg. This significant and inverse relationship between the two variables was also observed in group 2; with a daily increase in neonatal age, PAP reduced up to 0.25 mmHg. To put it another way, the gradual reduction of PAP in group 1 appeared to be slower than in group 2 (Graphic 2).

Although the effect of gestational age on SPAP reduction was seen to be greater than that of the neonatal age in both groups, the difference in group 1 was, however, more vivid and impressive (Tables 2, 3). Also, a meaningful and inverse relationship was identified between SPAP and neonatal weight in both groups; however, the relationship was markedly better in group 2 (Tables 2, 3).

In group 1, with a yearly rise in the maternal age, the PAP also increased up to 0.01 mmHg, showing no significant relationship between the maternal age and SPAP. A similar observation was made in group 2, where SPAP increased by 0.04 mmHg with a daily rise in the maternal age (Tables 2, 3).

In group 1, the mean SPAP proved to be 27.6+-4.4 mmhg and 28.4 + -4.1 mmhg in female and male neonates, respectively, but no significant difference was observed between the two genders (p=0.24). Similarly, in group 2, the mean SPAP appeared to be 22.4+-4.8 mmHg and 21.6+-5.4 mmHg in female and male neonates, respectively, but no significant difference was found between the two genders (p=0.34). On balance, the mean SPAP reached 24.86±1.5 mmHg and 25.24+/-5 mmHg in female and male neonates, respectively, so that no significant difference could be discerned between the two groups in terms of SPAP (p=0.54). The prevalence of PDA in both groups was 0.06% (i.e., one case in each group).

## Discussion

This cross-sectional study aimed at investigating the relationship between pulmonary arterial hypertension of neonates and IVF as an ART, the PAP in IVF neonates was found to be higher than that in the control group. In addition, the gestational and the neonatal age had a positive effect on the reduction of SPAP in the neonates after birth. Our study revealed PAP in IVF neonates to be 27.25% higher than that of the control group. In a study by Scherrer et al.^(9)^, the SPAP in the ART group was seen to be 30% higher than the control group. Although that study was conducted at an altitude of 3,450 meters and on a small population (65 ART children and 57 others in the control group) with a higher mean age (11.1 years in the ART group and 11.9 years in the control group), the ART and control groups had been matched in terms of gestational and neonatal age, hence approaching the results of ours.

However, the slight difference in the result might be due to the differences in the geography of the study setting, the sample size, and the mean age differences in the investigated populations.

Since all neonates were born through cesarean section and were matched on the basis of gestational and neonatal age, the only factor that brought about a significant difference in the PAP between the two groups in our study was seemingly the type of maternal pregnancy because maternal age failed to have an effect on the SPAP in both groups.

Perhaps, manipulating the embryos, that appear to be very sensitive to perturbations of the environment especially in their early stages of life during ART procedure, slows down the process of thinning of the pulmonary arterioles^(10)^. Moreover, the arterial stiffness, which is the main cause of increased systolic pressure, and recurrent periodic stress triggering the degeneration of the arterial wall^(11)^ seem to be more serious in ART neonates. In addition, neonates’ lungs are subject to vascular dysfunction^(12)^, which does not appear to be associated with parental factors, but to ART procedure^(13)^. Albeit, few human studies have been performed on ART neonates, it seems that ART induces early vascular dysfunction in lungs through epigenetic mechanisms accordingly contributing to pulmonary hypertension. This accords with what Mensah et al.^(14)^ reported regarding the effect of ART on vascular dysfunction in mice.

In our study, the gestational age was also proved to have a positive effect on SPAP reduction so that with an increase in the gestational age, SPAP subsided, as the rise in gestational age is associated with an increase in the soluble guanylate cyclase (sGC) function in the lungs^(15)^.

This substance acts as an important receptor for nitric oxide in the pulmonary vessels and relaxes the vascular smooth muscles^(14,15)^. In Mensah et al.^(14)^ study, it was demonstrated that the level of sGC mRNA, which acts as a mediating agent for the NO function on the differentiation of the smooth muscles of neonates’ pulmonary vasculature, appears to be low in sheep fetus (preterm gestation of 126 days), however, it heightens significantly during the late preterm and early term gestation (137 days)^(14,16)^. Furthermore, in the Sprague-Dawley rats, the sGC activity in the lungs in the late gestation and the early newborn period proved to be high^(14)^.

Gonadotropins like Gonal-F, used by the mother to induce pregnancy through the placenta, may be transmitted to the bloodstream of the fetus and trigger thrombotic events in the pulmonary vasculature. This is similar to what occurs in the mother’s pulmonary arteries^(17,18,19)^ and provides the underlying reason for maintaining pulmonary hypertension in the afterbirth period. Mothers’ primary hormone disturbances, including polycystic ovary syndrome, which contribute to infertility and pulmonary thromboembolism in the fetus, may also engender this problem as the risk of such incidents is high in such mothers^(20)^. Moreover, it was identified that with an increase in the neonatal age, SPAP dwindles away. This is due to the fact that the gradual thinning of the muscular membrane, dilatation of the pulmonary arteries, the growth of the existing arteries, and the formation of new arteries all tend to occur during the postnatal pulmonary development that lasts for weeks and months, and gradually exert an influence on the reduction of PVR and, consequently, SPAP^(1)^. In our study, only one case affected by PDA was observed in each group. As all the subjects were at term and revealed no effective factors for their arterial duct to be kept open^(17,19)^, the prevalence proved to be very low in at-term neonates (approximately 2,000-2,500 live births)^(18)^.

Of interest, although in our study, SPAP appeared to be higher in the IVF group than the control and the difference was significantly higher in the former, only two neonates were affected with mild pulmonary hypertension (systolic pressure of 37 and 38 mmHg) and two with pressure leveled at 36 mmHg. Due to the low prevalence of the case (approximately 2 per 1,000 live births), more investigations are needed to diagnose neonates affected with pulmonary hypertension.

## Conclusion

In the current study that was about PAP in at-term IVF neonates based on a cross-sectional study, linear regression between SPAP and gestational age in the two groups illustrates a daily boost in the gestational age, and intrauterine life significantly impinging on the reduction of SPAP in postnatal period; it follows that it seems to be unwise to give birth to IVF neonates too early. Additionally, the comparison of linear regression between SPAP and the neonatal age in both groups indicates that gradual decrease of the PAP in group 1 proves to be slower than in group 2 (-0.19 vs -0.25). In other words, the pulmonary artery wall thinning proceeds less gradually in group 1.

Further, our study regards IVF as an predisposing factor in the incidence of pulmonary arterial hypertension in neonates. Therefore, it is recommended that IVF neonates be monitored for pulmonary hypertension during the neonatal period to receive appropriate and timely treatment.

However, more studies and larger sample sizes are needed to comprehensively address and capture the causes of pulmonary hypertension in IVF neonates.

## Figures and Tables

**Table 1:**
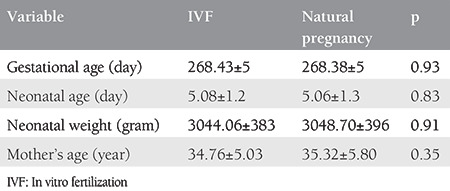
Demographics of mother and neonate in terms of type of fertilization

**Table 2:**
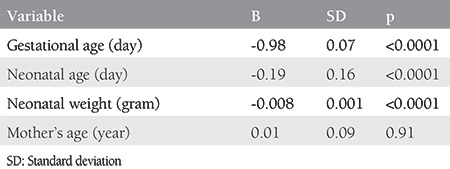
Linear regression analysis of mother’s gestational age, neonatal age and weight, and mother’s age in the in vitro fertilization group

**Table 3:**
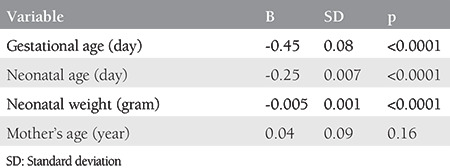
Linear regression analysis of mother’s gestational age, neonatal age and weight, and mother’s age in the natural pregnancy group

**Graphic 1:**
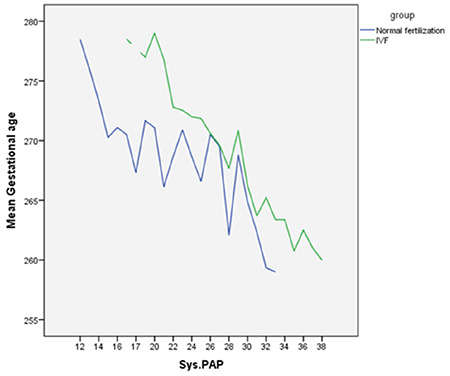
Comparison of the linear regression of in vitro fertilization group and control group based on systolic pulmonary artery pressure and gestational age IVF: In vitro fertilization, SPAP: Systolic pulmonary artery pressure

**Graphic 2:**
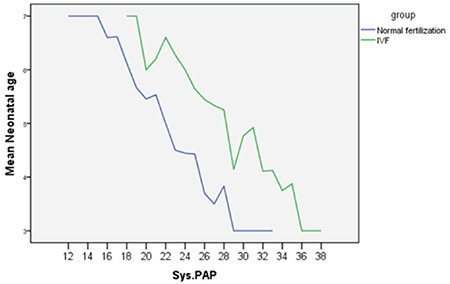
Comparison of the linear regression of in vitro fertilization group and control group based on systolic pulmonary artery pressure and neonatal age IVF: In vitro fertilization, SPAP: Systolic pulmonary artery pressure
